# Incisional hernia prevention in high-risk midline laparotomy using onlay PVDF mesh: multicenter prospective observational study

**DOI:** 10.3389/jaws.2026.17201

**Published:** 2026-09-28

**Authors:** Jose Luis Rodicio Miravalles, Javier López Monclús, Sonia Amoza Pais, María Moreno Gijón, Antonio López López, Natalia Gutiérrez Corral, Luis Alberto Blázquez Hernando, Rafael Menéndez de Llano Ortega, María Isabel Diez Pérez de las Vacas, Lourdes Sanz Álvarez, Miguel Angel Garcia-Urena

**Affiliations:** 1 Division of General Surgery, Hospital Universitario de Cabueñes, Gijón, Spain; 2 Division of General Surgery, Hospital Universitario Puerta de Hierro Majadahonda, Madrid, Spain; 3 Division of General Surgery, Hospital Universitario Central de Asturias, Oviedo, Spain; 4 Division of General Surgery, Hospital Universitario Nuestra Señora del Prado, Toledo, Spain; 5 Division of General Surgery, Hospital Universitario San Agustin, Avilés, Spain; 6 Division of General Surgery, Hospital Universitario Ramón y Cajal, Madrid, Spain; 7 Radiology Department, Hospital Universitario Central de Asturias (HUCA), Oviedo, Spain; 8 Radiology Department, Hospital Universitario del Henares, Madrid, Spain; 9 Division of General Surgery, Faculty of Medicine, Universidad Francisco de Vitoria, Hospital Universitario del Henares, Madrid, Spain

**Keywords:** high-risk patients, incisional hernia, midline laparotomy, polyvinylidene fluoride (PVDF), prophylactic mesh, emergency surgery

## Abstract

**Introduction:**

Prophylactic mesh reinforcement reduces the risk of incisional hernia after midline laparotomy in high-risk patients, although concerns regarding complications and implementation limit its routine use. This multicentre prospective observational study evaluated long-term outcomes after onlay polyvinylidene fluoride (PVDF) mesh reinforcement in high-risk patients undergoing midline laparotomy in elective and emergency settings.

**Materials and methods:**

A prospective multicentre observational cohort study was conducted in tertiary and secondary hospitals within the Spanish National Health Service between July 2016 and June 2022. Adult patients undergoing midline laparotomy with more than one risk factor for incisional hernia were included. Abdominal wall closure was reinforced with an onlay PVDF mesh visible on MRI. The primary outcome was incisional hernia at 2 years. Secondary outcomes included mesh-related complications and factors associated with incisional hernia.

**Results:**

A total of 180 patients were analysed. Mean age was 64.6 years and mean BMI was 27.4 kg/m^2^. Most procedures were performed for oncological disease (72.8%), 32.8% were emergency operations, and 23.9% were performed in contaminated or dirty surgical fields. Surgical-site infection occurred in 12.8% and seroma in 9.4% during index admission. Mesh removal was required in four patients (2.2%). Two-year follow-up among surviving patients reached 96.1%. In the intention-to-treat analysis, incisional hernia developed in 21 patients (11.3%), compared with 16 patients (8.9%) in the per-protocol analysis. BMI (OR 1.19, 95% c.i. 1.05 to 1.33; P = 0.008) and stoma formation (OR 5.77, 95% c.i. 1.75 to 19.30; P = 0.004) were independently associated with incisional hernia. MRI demonstrated no significant mesh contraction.

**Conclusion:**

In this prospective multicentre observational study, prophylactic onlay PVDF mesh reinforcement in high-risk midline laparotomy, including emergency and contaminated procedures, was feasible and associated with a low incidence of incisional hernia and acceptable mesh-related morbidity. These findings are hypothesis-generating and warrant further evaluation in controlled studies.

## Introduction

Incisional hernia (IH) is one of the most frequent complications encountered in daily surgical practice. It carries a substantial impact on healthcare system resources, contributes to loss of work productivity, diminishes quality of life, negatively affects body image, and is associated with pain, risk of emergency surgery, and impaired abdominal wall function [1–3]. Given this considerable burden, effective prophylaxis must be regarded as a fundamental pillar in the comprehensive management of patients undergoing abdominal surgery [4, 5].

Current clinical guidelines and meta-analysis acknowledge prophylactic mesh placement (PMP) as a safe and effective preventive intervention and advocate its consideration in patients at elevated risk for incisional hernia development [6–12]. High-quality evidence has demonstrated that, in the context of midline laparotomy, PMP significantly reduces the incidence of incisional hernia among high-risk populations, particularly in individuals with obesity and in those undergoing abdominal aortic aneurysm repair.

Despite strong evidence supporting PMP, uncertainty about its risk–benefit profile limits widespread adoption. Both surgeons and patients remain concerned about implanting mesh in the absence of a hernia, particularly regarding potential complications such as infection, seroma, or chronic pain [13–15]. Additionally, the perception that PMP prolongs operative time, together with the uncertain risk of mesh-related events, further discourages routine use and raises concerns about operating room efficiency and healthcare resource utilization [16].

Imaging is an essential adjunct in the diagnosis of incisional hernia and is increasingly important for clinical decision-making. In this study, imaging enabled assessment of abdominal wall healing and detection of potential adverse events not apparent on physical examination.

In view of the ongoing debate surrounding the advantages and potential drawbacks of mesh reinforcement, together with the challenges associated with its implementation in routine practice by general surgeons, the present multicentre study was designed to assess the use of polyvinylidene fluoride (PVDF) mesh and to adopt a widely generalizable mesh position across hospitals of varying complexity and in high-risk surgical patients and settings, including emergency procedures and contaminated fields. Our objective was to address the persistent uncertainties in this area, with particular emphasis on long-term outcomes related to the prevention of incisional hernia.

## Materials and methods

This was a prospective multicentre observational cohort study. The study report was written following the Strengthening the Reporting of Observational Studies in Epidemiology (STROBE) statement [17].

### Setting

All of the participating centres belonged to the Spanish National Health Service and had at least one surgeon with expertise in abdominal wall surgery. The surgeons involved in the study were general surgeons who did not necessarily specialize in abdominal wall surgery. A website was available to them where they could find indications and an educational video of the technique, as well as support for registering patients, in accordance with the national data protection laws. The surgeons were in contact via email and a social media group (WhatsApp) in case of incidents during the study period, none of which included the personal data of the participants. All of the centres had an abdominal radiologist of reference to cite and evaluate the magnetic resonance imaging (MRI). The study was approved by the Research Ethics Committee (CEIm) of Hospital Nuestra Señora del Prado (approval code 17/16). This approval covered all participating centres within the Spanish National Health System, and no additional site-specific ethics approvals were required. The study started in July 2016 under the name of the VISIBLE study, and the protocol was registered at ClinicalTrials.gov (NCT03105895). Written informed consent was obtained from all participants or, where applicable, from their legal representatives.

### Participants

#### Inclusion criteria

The included patients needed to have more than one risk factor for IH: age greater than 60 years old, body mass index (BMI) > 30 kg/m^2^, diabetes, chronic obstructive pulmonary disease, heart disease, smoking, kidney disease, neoplasia, liver disease, immunosuppression or an emergency operation.

#### Exclusion criteria

Patients under 18 years of age, previous IH or midline laparotomy, life expectancy under 12 months, haemodynamic instability during surgery, refusal to sign the informed consent, simultaneous participation in another study that interfered with the intervention and/or the results, an iron allergy, or contraindications for MRI (i.e., non-MRI-compatible devices, patients with claustrophobia) were excluded.

#### Follow-up

A complete postoperative follow-up was performed at 6 weeks and 3, 6, 12, 18 and 24 months after surgery. The collected data were entered on the study web page, focusing on adverse events related to the abdominal wall. Follow-up included physical examinations and 6-week postoperative MRI. An additional MRI was planned for the 12-month follow-up. Additional data from the follow-up of the underlying disease were collected and reviewed, including imaging studies performed for their primary pathology that could contribute to the diagnosis of incisional hernia. Incisional hernia was diagnosed based on both clinical examination and available radiological findings during follow-up.

#### Imaging technique

Imaging sequences were obtained using a single shot fast spin echo sequence with suspended respiration as a locator. T1 echo gradient sequences were utilized in the three planes of the space to assess the meshes using paramagnetic susceptibility in these sequences. Finally, a T2 fast spin echo sequence was performed with respiratory synchronization for anatomical assessment and evaluation of any possible complications.

#### Mesh features

DynaMesh®-CICAT visible (FEG Textiltechnik, Aachen, Germany) was investigated in this study. This mesh was manufactured exclusively for the purposes of this study. Its characteristics include: length: 35 cm, width: 7.5 cm. Composite: 90% PVDF, 10% PVDF + incorporated triiron tetraoxide (Fe_3_O_4_ <1% (w/w)). Weight: ∼131 g/m^2^ (∼67 g/m^2^ PVDF-Polypropylene equivalent); mean thickness: 0.75 mm; mean pore size: 2.6 mm.

#### Surgical technique

Closure of the abdominal wall was performed with a running suture with a long-term absorbable synthetic monofilament suture USP number 0 or 1 in 1 plane, spaced 0.5–1 cm apart and from the cut edge. The 4:1 suture length–to–wound length ratio for abdominal wall closure was recommended; however, it was not formally recorded. Dissection of the prefascial space was performed by detaching the subcutaneous cellular tissue of the aponeurosis up to 4 cm on both sides and 2 cm at the ends of the incision. The umbilicus was detached from its scar on the linea alba. The length of the incision was measured at the end of the closure of the linea alba. A PVDF mesh was then placed in the onlay prefascial position, with an overlap of 2 cm at each extreme, fixing the edges with interrupted stitches of USP 2/0 absorbable synthetic monofilament separated from each other by 3–4 cm. The subcutaneous cellular tissue was approximated by interrupted braided synthetic absorbable multifilament suture USP 2/0 fixed to the mesh to reduce dead spaces. The umbilical scar was also fixed to the mesh. A 10–12 Fr aspiration drainage tube was placed on the mesh and was removed between the third and fifth postoperative days. The skin was closed with staples. No additional special care was taken with the surgical wound during the postoperative period [18].

### Outcomes

The primary endpoint was to evaluate the incidence of incisional hernia at 2 years of follow-up, as part of this long-term study assessing the onlay use of a mesh for which evidence regarding its role in incisional hernia prevention remains limited.

Secondary endpoints included the analysis of risk factors for incisional hernia development and mesh-related adverse events.

### Variables

The patient characteristics and intraoperative and postoperative data collection were focused on the incisional hernia risk factors and adverse events related to the pathology of the abdominal wall after surgery. The classification of the surgical site infection (SSI) was performed using the Centers for Disease Control (CDC) classification guideline [19]. Data were recorded in an online database as it was collected.

### Sample size

The VISIBLE study was designed to enrol 200 patients, based on a sample size calculation assuming a 15% incidence of postoperative wound events, with a precision of ±5% and a 95% confidence level.

### Statistical analysis

All statistical analyses were performed using R 4.5.0 statistical software (R Foundation for Statistical Computing, Vienna, Austria). Categorical variables are described in frequency tables, and quantitative variables are summarized using measures of central tendency and dispersion. Both univariable and multivariable logistic regression models were constructed to estimate odds ratios for candidate variables potentially associated with incisional hernia (considered a binary outcome). Candidate variables for multivariable analysis were selected based on clinical relevance. Backward variable reduction was subsequently performed using the Akaike Information Criterion (AIC). Given the limited number of outcome events, Firth penalised logistic regression and bootstrap internal validation were performed to assess potential small-sample bias, variable-selection stability, discrimination and calibration.

For time-to-event analysis, death before incisional hernia was considered a competing event. Cumulative incidence functions were used to estimate the probability of incisional hernia over time. Gray’s test was used to compare cumulative incidence between stoma groups, and Fine–Gray regression was used to estimate subdistribution hazard ratios. A p-value <0.05 was considered statistically significant.

## Results

### Demographic characteristics

The initial sample comprised 200 patients assessed for eligibility between July 2016 and June 2022. After exclusion of 15 patients, 185 were included in the study. Following further exclusion of 5 patients due to abdominal wound dehiscence, the final sample size for analysis was 180 patients (Figure 1). Of these, 63.9% were male, with a mean age of 64.6 years and a median BMI of 27 kg/m^2^. The remaining variables are presented in Table 1.

**FIGURE 1:**
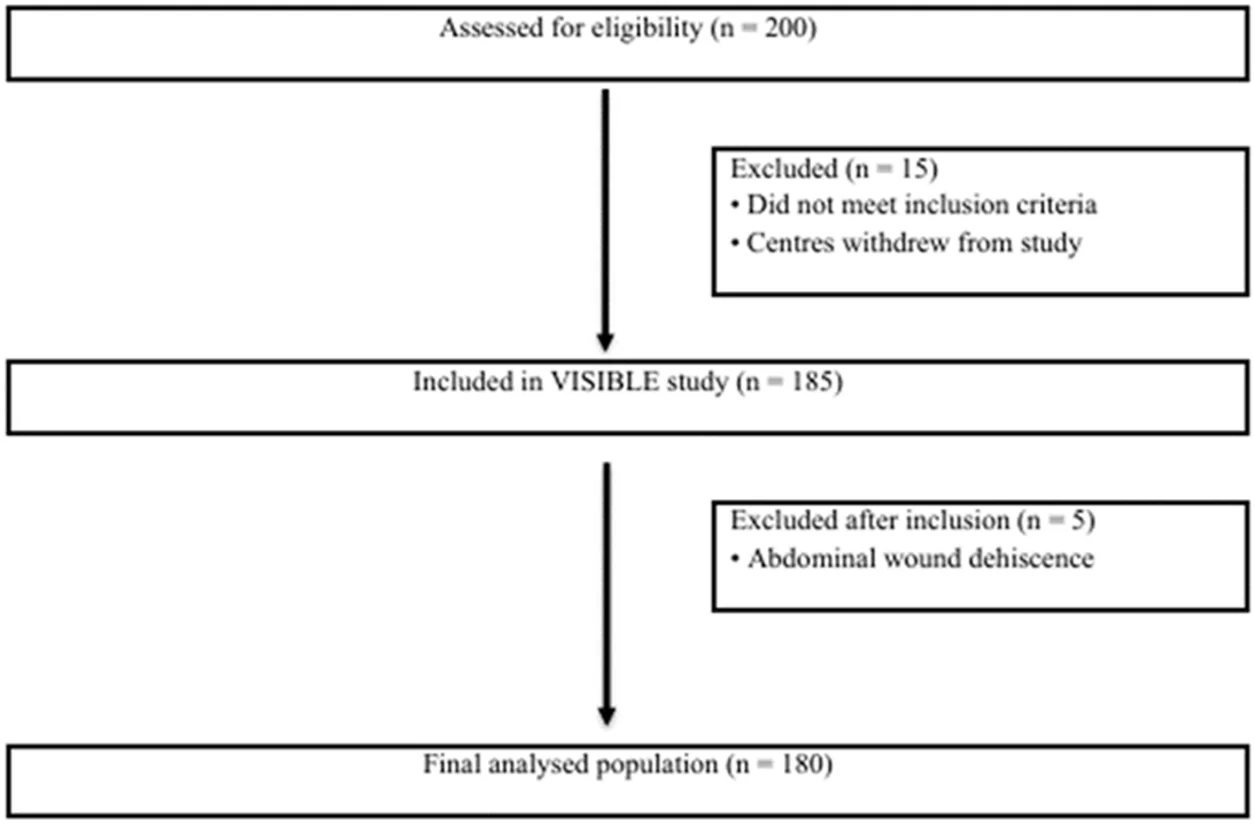
Flow diagram of the Visible observational study.

**TABLE 1:** Baseline patient characteristics^a^.

Variable (n = 180)	Value
Age, years, mean (SD), range	64.6 (13.3), 22–93
Gender: Male/female	115 (63.9)/65 (36.1)
BMI, kg/m^2^, mean (SD), range	27.4 (4.9), 18–42.5
Hospitals <500 beds >500 beds	100 (55.6) 80 (44.4)
Smoking Non-smoker Smoker Ex-smoker	81 (45) 42 (23.3) 57 (31.7)
Hypertension	76 (42.2)
DM	34 (18.9)
Neoplasia	133 (73.9)
Immunodepression	19 (10.6)
COPD	27 (15)
Heart disease	27 (15)
Malnutrition	14 (7.8)
Liver disease	8 (4.4)
Kidney disease	9 (5)
Coagulopathy	5 (2.8)
Connective tissue disorders	2 (1.1)

^a^ Values are presented as the No. (%) of patients unless otherwise specified.

Abbreviations: BMI, body mass index; DM, diabetes mellitus; COPD, chronic obstructive pulmonary disease.

### Characteristics of the surgery

The majority of procedures were elective (67.2%), performed for oncologic pathology (72.8%), and classified as clean-contaminated (68.3%). A stoma was required in 15% of patients. The median operative time was 178 min, the median incision length was 20 cm, and the median length of hospital stay was 10 days. These results are detailed in Table 2.

**TABLE 2:** Characteristics of admission and surgery^a^.

Indication: emergency/elective	59 (32.8)/121 (67.2)
Aetiology Tumour Inflammation Bleeding Adhesions Other	131 (72.8) 34 (18.9) 3 (1.7) 4 (2.2) 8 (4.4)
Type of surgery Lower GI tract Upper GI tract Trauma Hepatobiliary Other	132 (73.7) 36 (20.1) 1 (0.6) 5 (2.8) 6 (3.4)
Surgical field Clean Clean-contaminated Contaminated Dirty	14 (7.8) 123 (68.3) 23 (12.8) 20 (11.1)
Surgical time in minutes, mean (SD), range	187 (78.2) 65–570
Incision length in cm, mean (SD), range	20.4 (3.9) 10–31
Ostomies No Ileostomies Colostomies	153 (85) 12 (6.7) 15 (8.3)
Length of hospital stay in days, mean (SD), range	12.4 (8.4) 4–56
Intra-operative complications	6 (3.3)
Blood transfusion	37 (20.6)

^a^ Values are presented as the No. (%) of patients unless otherwise specified.

Postoperative outcomes are summarized in Table 3, highlighting a 4.4% rate of septic shock. Among surgical complications, the incidence of SSI was 12.8%, seroma 9.4%, and anastomotic dehiscence 10%.

**TABLE 3:** Postoperative outcomes^a^.

Medical complications Cardiac Respiratory Renal UTI Septic shock	7 (3.9) 7 (3.9) 5 (2.7) 2 (1.1) 8 (4.4)
Surgical complications SSI, no (%) Superficial Deep Organ or body space Seroma Haematoma Exposure of mesh Ileus Anastomotic dehiscence	23 (12.8) 10 (5.5) 5 (2.7) 8 (4.4) 17 (9.4) 11 (6.1) 2 (1.1) 31 (17.2) 18 (10)
Subsequent operation (reintervention)	11 (6.1)
Mesh removal	4 (2.2)
In-hospital mortality	4 (2.2)

Abbreviations: UTI, urinary tract infection; SSI, surgical site infection.

^a^ Values are presented as the No. (%) of patients unless otherwise specified.

Reoperation was required in 11 patients. In seven patients, mesh removal was not necessary; in one of these, the complication was limited to the subcutaneous plane, whereas in the remaining six the abdominal cavity was accessed through the mesh. In three patients, the mesh was replaced due to contamination of the surgical field, and in the final patient the mesh was removed without replacement because of poor clinical prognosis; this patient subsequently died. Overall, four patients (2.2%) died during the index hospitalization.

### Patient inclusion

Overall, 55.6% of patients underwent surgery in hospitals with fewer than 500 beds, with Hospital Universitario del Henares contributing the largest proportion of cases (42.8%). Figure 2 illustrates patient inclusion by hospital and by year, as well as the year each center joined the study.

**FIGURE 2:**
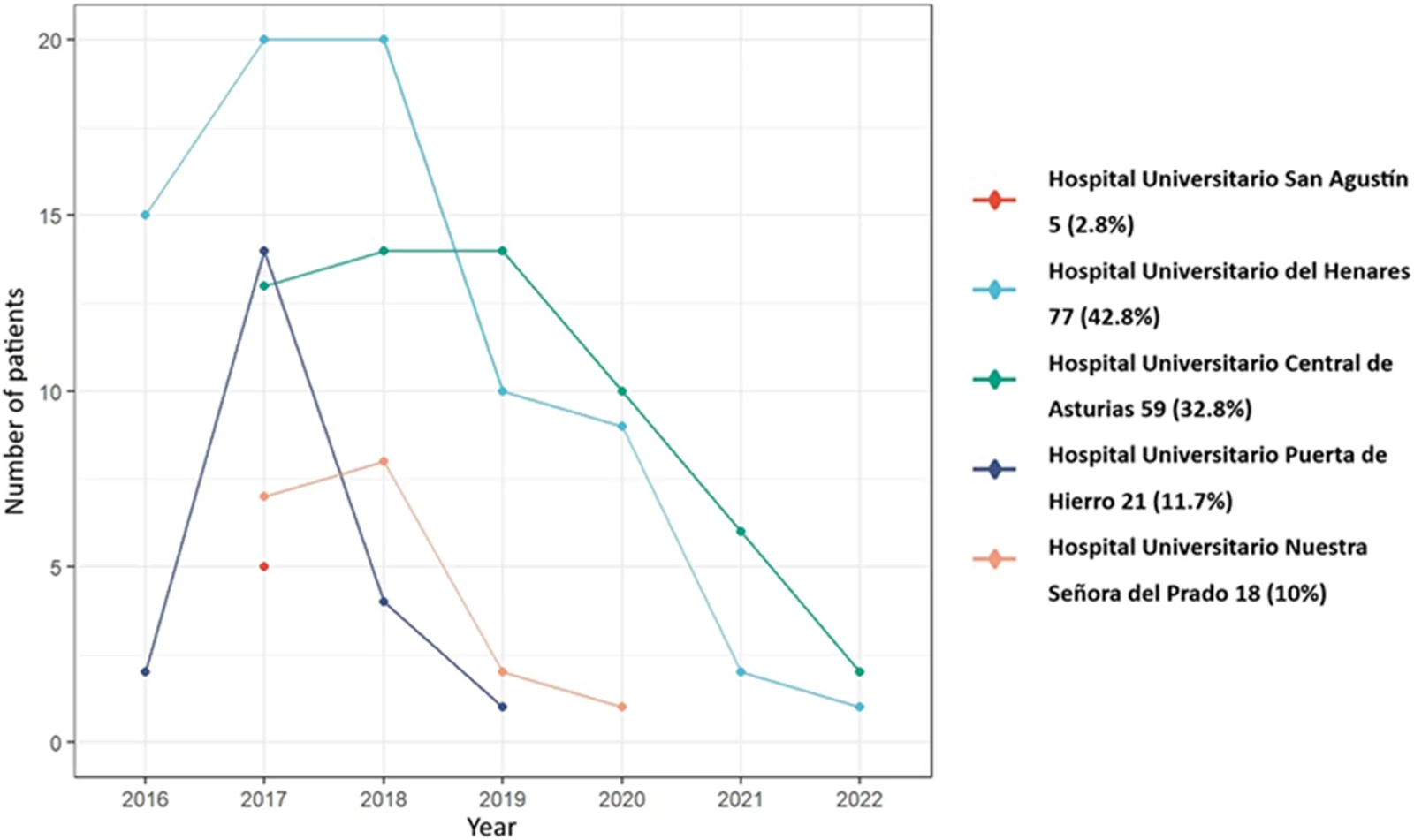
Patient inclusion by hospital and year of centre participation.

### Follow-up

At 1 year, follow-up data were available for 168 patients. After accounting for the 10 patients who had died, the follow-up rate among survivors was approximately 99%, decreasing to 96.1% at 2 years. All deaths were observed in patients who had undergone surgery for oncologic pathology (p = 0.0134), and none were considered related to the prophylactic mesh.

### Abdominal wall complications

Abdominal wall surgery–related complications are summarized in Table 4. Six-week outcomes were supported by MRI, performed in 136 patients (78.1%). The incidence of seroma increased from 9.4% during the index hospitalization to 20.7% at 6 weeks, largely due to MRI detection; at 3 months, only four patients (2.4%) had persistent seroma. Superficial SSI occurred in 5.5% during hospitalization, 2.3% at 6 weeks, and in three patients (1.8%) at 3 months; one case failed conservative management and required mesh removal at 2 years during incisional hernia repair. Mesh exposure was managed conservatively in two patients (1.2%) at 3 months. One enterocutaneous fistula occurred in the context of infection treated with negative-pressure wound therapy, requiring reoperation with intestinal resection and repair of the incisional hernia and onlay mesh. No significant differences in complications were observed according to hospital bed capacity. At 3 months, mean laparotomy pain was 0.2 on the visual analogue scale; thereafter, pain was not formally assessed, and no cases of chronic pain or need for specific treatment were identified.

**TABLE 4:** Follow-up outcomes^a^.

Outcome	During hospital stay n 180	6 weeks n 174	3 months n 169	6 months n 169	12 months n 168	18 months n 160	24 months n 156	Total
Seroma	17 (9.4)	36 (20.7)	4 (2.4)	-	-	-	-	​
Wound infection	10 (5.5)	4 (2.3)	3 (1.8)	1	1	1	1	10/180 (5.5)
Exposure of mesh	2 (1.1)	6 (3.4)	2 (1.2)	-	-	-	-	​
Incisional hernia	-	-	2 (1.2)	2 (1.2)	7 (4.1)	2 (1.2)	3 (1.9)	16/180 (8.9)
Mortality	4 (2.2)	-	-	1 (0.6)	5 (2.9)	4 (2.5)	3 (1.9)	17/180 (9.4)
MRI	-	136 (78.1)	-	-	110 (65.1)	-	-	​
CT scan	-	-	-	-	113 (66.9)	-	95 (60.1)	​

Abbreviations: MRI: Magnetic resonance imaging. CT: computed tomography.

^a^ Values are presented as the No. (%) of patients unless otherwise specified.

### Imaging study

MRI was performed in 136 patients (78.1%) at 6 weeks and in 110 patients (65.1%) at 1 year. Reasons for not undergoing MRI included non-compatible pacemakers and previously unrecognized claustrophobia, as well as reluctance among older patients and cases of death or unfavorable clinical course. In the follow-up period, pandemic-related conditions limited the performance of imaging studies, which were prohibited in some centers.

A total of 100 patients underwent MRI at both time points. In this subgroup, the mean mesh length was 20.3 cm (SD = 3.9) at 6 weeks and 19.9 cm (SD = 3.8) at 1 year, while the mean width was 7.02 cm (SD = 1) and 7.04 cm (SD = 1.1), respectively, with no statistically significant changes observed over time. Representative MRI images illustrating the visibility and appearance of the iron oxide–loaded PVDF mesh during follow-up are shown in Figure 3.

**FIGURE 3:**
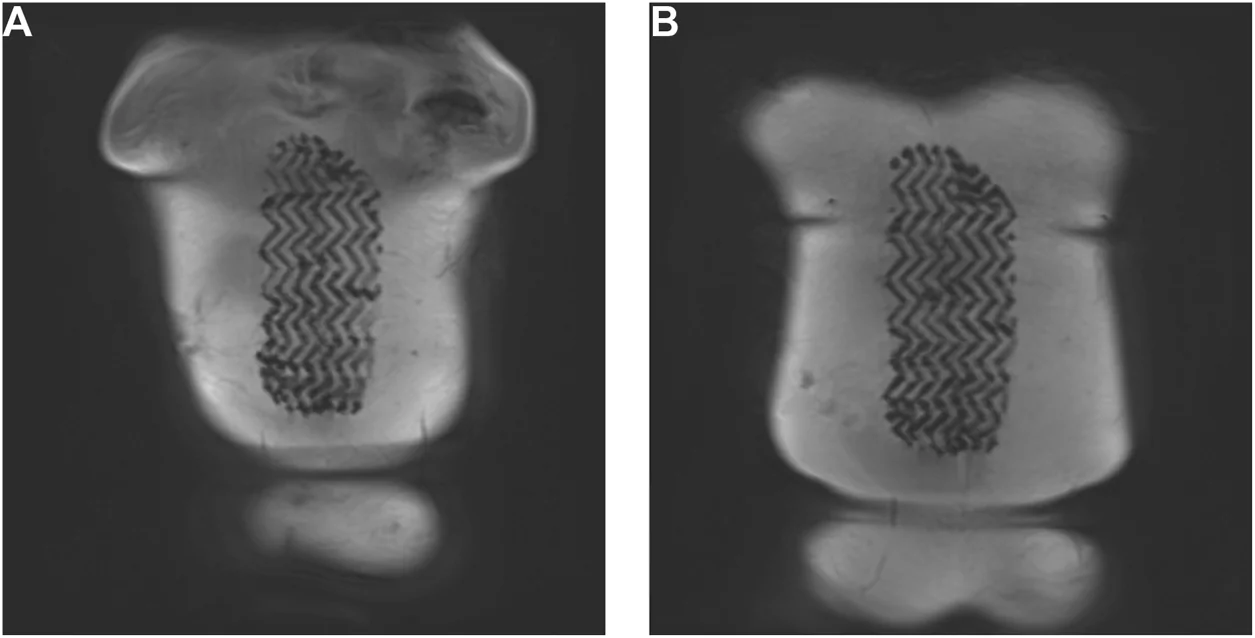
Representative MRI images demonstrating the visibility of the iron oxide–loaded PVDF mesh. **(A)** Six-week postoperative MRI. **(B)** One-year postoperative MRI.

At 1 year, CT scans had been performed in 113 patients (66.9%), and at 2 years in 95 patients (60.1%), as part of the follow-up of their underlying conditions; these imaging studies were subsequently reviewed to support the assessment of abdominal wall integrity. At 1 year of follow-up, 23 patients (13.7%) have no imaging studies performed.

### Follow-up incisional hernia

During the 2-year follow-up, the overall incidence of incisional hernia was 11.3% according to the intention-to-treat analysis. In the per-protocol analysis, excluding patients with early postoperative wound dehiscence, 16 incisional hernias were diagnosed (8.9%), with the highest incidence observed at the 1-year assessment. When the cohort was chronologically divided into the first 90 and the subsequent 90 patients included in the series, 75% of incisional hernias were observed during the initial period. Comparison between these two groups showed that emergency surgery was the only significantly different variable, accounting for 43.3% of procedures in the first group versus 22.2% in the second (p = 0.004).

With respect to the management of patients who developed incisional hernia, seven underwent hernia repair concomitantly with another procedure, most commonly during restoration of bowel continuity or due to progression of oncological disease. Three patients underwent elective hernia repair, including one case associated with chronic *Pseudomonas aeruginosa* mesh infection. Of the six patients who did not undergo repair, two died from oncological disease. Two patients remain under follow-up. The lasting two patients were radiological (MRI-detected) incisional hernias without clinical or physical findings; notably, these were the only patients in the hernia group without underlying malignancy.

The presence of superficial or deep surgical site infection was associated with an increased risk of incisional hernia compared with no infection; however, this did not reach statistical significance (OR 2.92, 95% c.i. 0.61 to 10.71; p = 0.129).

Figure 4 presents the overall cumulative incidence of incisional hernia, whereas Figure 5 presents cumulative incidence functions according to stoma status.

**FIGURE 4:**
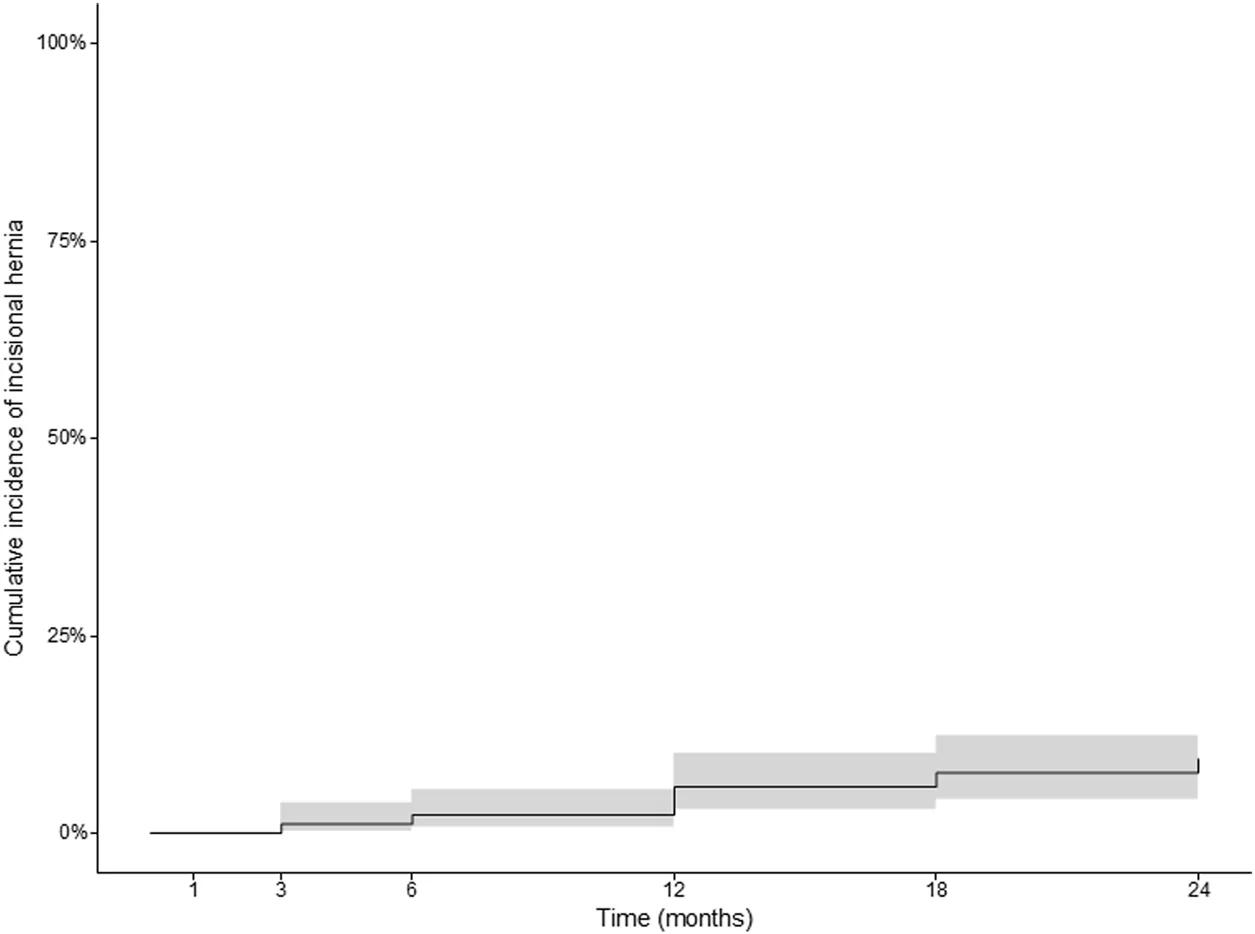
Cumulative incidence function of incisional hernia accounting for death as a competing event.

**FIGURE 5:**
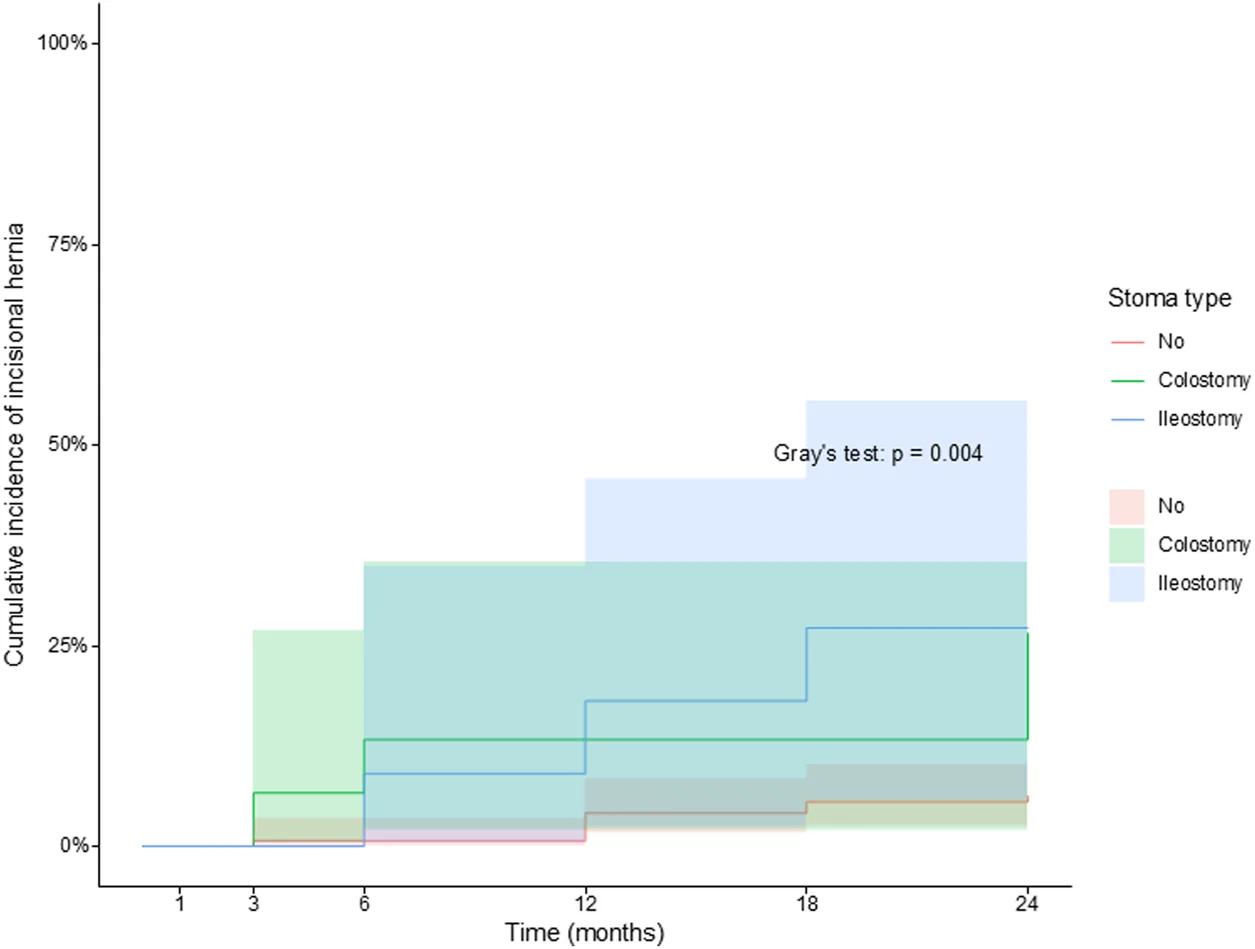
Cumulative incidence function of incisional hernia according to stoma type, accounting for death as a competing event.

At 24 months, the cumulative incidence of incisional hernia was 6.3% in patients without a stoma, 26.7% in those with a colostomy, and 27.3% in those with an ileostomy. Gray’s test showed a significant difference in cumulative incidence between stoma groups (p = 0.004). In the Fine–Gray model, both colostomy (sHR 4.67, 95% CI 1.49–14.6; p = 0.008) and ileostomy (sHR 4.85, 95% CI 1.35–17.4; p = 0.015) were associated with a higher subdistribution hazard of incisional hernia compared with no stoma.

### Logistic regression model

In univariable analysis, colostomy (OR 5.82, 95% c.i. 1.40 to 21.27; p = 0.009), ileostomy (OR 5.33, 95% c.i. 1.05 to 21.86; p = 0.026), and BMI (OR 1.12, 95% c.i. 1.01 to 1.24; p = 0.032) were associated with the development of incisional hernia (Table 5).

**TABLE 5:** Variables related to incisional hernia. Univariable analysis.

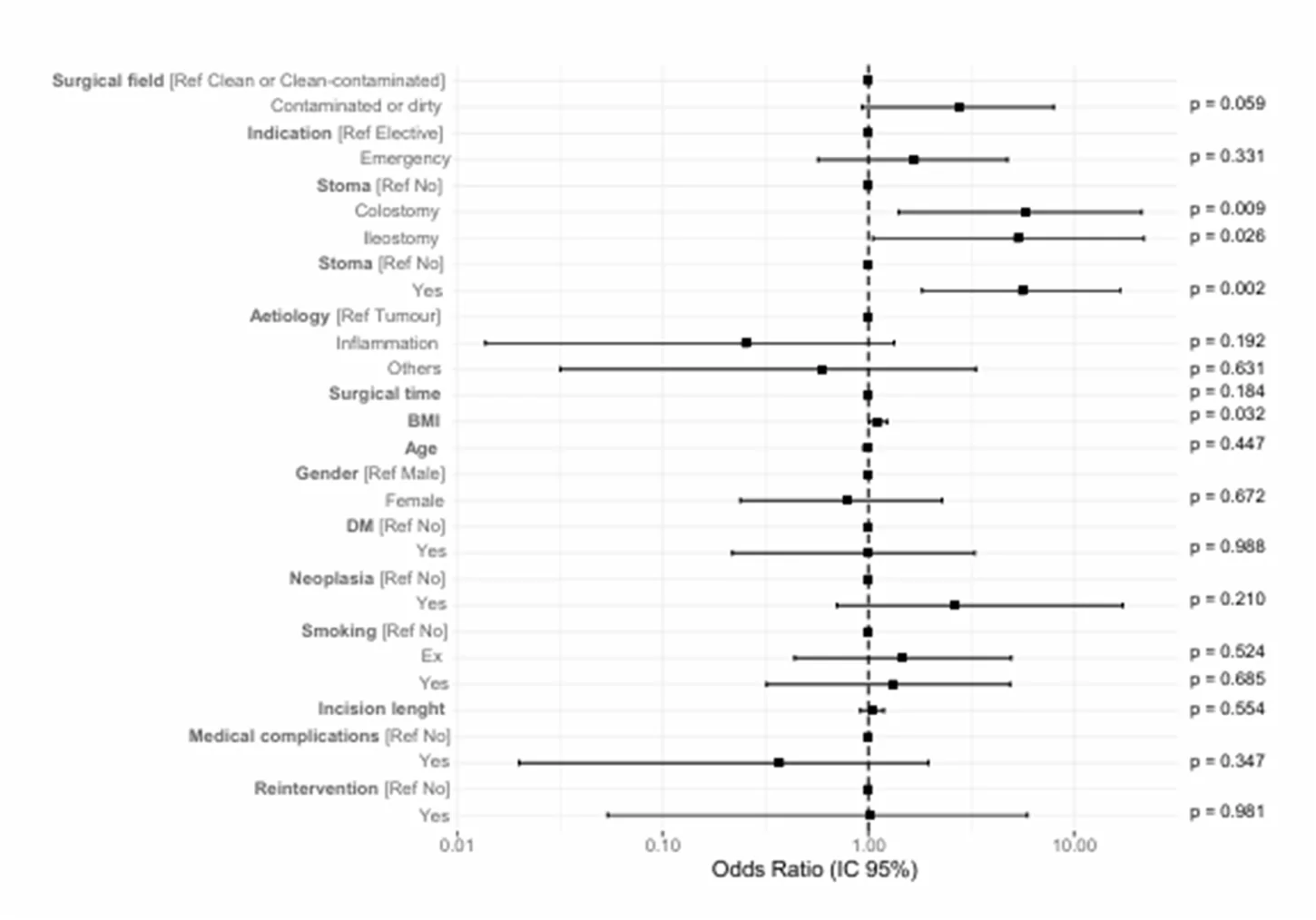

Abbreviations: BMI, body mass index; DM, diabetes mellitus.

The final multivariable model included four predictors: stoma, operative duration, BMI, and neoplasia, corresponding to an events-per-variable ratio of 4. Longer operative time was associated with a lower risk of incisional hernia (OR 0.99, 95% c.i. 0.97 to 0.99; p = 0.023), whereas higher BMI (OR 1.19, 95% c.i. 1.05 to 1.33; p = 0.008) and the requirement for a stoma (OR 5.77, 95% c.i. 1.75 to 19.30; p = 0.004) were associated with higher odds of incisional hernia (Table 6).

**TABLE 6:** Variables related to incisional hernia. Multivariable analysis.

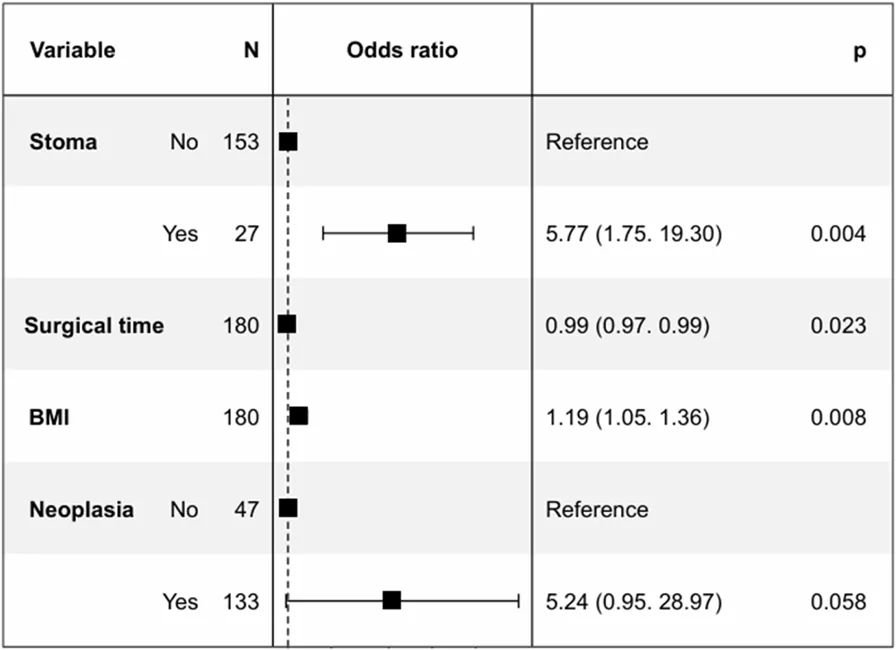

Abbreviations: BMI, body mass index.

The Firth penalised model yielded similar estimates to the conventional logistic regression model. Bootstrap internal validation yielded an apparent AUC of 0.811 (95% CI 0.690–0.932), an estimated optimism of 0.033, and an optimism-corrected AUC of 0.778. The optimism-corrected calibration slope was 0.784.

## Discussion

Prophylactic mesh reinforcement has been associated with a lower incidence of incisional hernia in high-risk patients undergoing midline laparotomy in previous studies. Under this premise, the present multicentre observational study evaluated the feasibility and outcomes of a standardized protocol for prophylactic mesh placement under real-world clinical conditions across diverse hospital settings, regardless of underlying pathology, the subspecialization of the general surgeon, the degree of surgical field contamination, and whether the procedure is performed in an elective or emergency context.

Of the initially estimated study sample, 15 patients are excluded for not meeting the inclusion criteria. Five patients who present with early postoperative abdominal wound dehiscence are excluded from the long-term analysis, as the primary objective is to evaluate the sustained outcomes of prophylactic mesh placement. This approach is further supported by the subsequent clinical course of these cases: three patients die shortly after surgery, one is managed conservatively and later dies during follow-up, and one requires reoperation with replacement of the original mesh by a different type. Under these circumstances, inclusion of these cases is considered likely to introduce substantial confounding and not to contribute meaningfully to the assessment of incisional hernia development related to prophylactic mesh, thereby potentially obscuring the long-term effects under investigation.

The inclusion of patients across the participating centres followed a similar pattern, with higher recruitment during the first year followed by a progressive decline over the study period, a finding commonly observed in long-term multicentre prospective studies [20, 21]. It may also reflect the limited adoption of prophylactic mesh in many general surgery departments, where its use remains a practice with minimal spillover across surgical services [15, 16].

The characteristics of the series reflect a high-risk patient population, with a median age of 66 years, a median body mass index of 27 kg/m^2^, and a predominance of oncologic pathology (72.8%). This profile implies not only an increased risk of complications during hospitalization but also during follow-up, given the potential need for additional surgical procedures. Clinical contexts are likewise high risk, with 32.8% of cases performed under emergency conditions and 23.9% classified as contaminated or dirty surgery, both settings in which the systematic use of prophylactic mesh remains controversial [22]. Overall, these features represent a cohort highly representative of the complex case mix encountered in general surgery departments.

As previously reported in the short-term outcomes [23], in patients requiring reintervention, the presence of a prophylactic mesh represents an additional technical consideration; however, in most cases, access to the abdominal cavity is achieved through the mesh without major difficulty. Overall, the use of prophylactic mesh does not result in significant complications in patients requiring reoperation in either the short or long term, a finding consistent with previous studies [24], and therefore should not be regarded as a deterrent or justification for omitting its use in high-risk patients [13].

As expected with onlay PMP, seroma is relatively frequent in the early postoperative period, particularly when assessed by MRI, which likely leads to overdiagnosis, as most cases are asymptomatic and not detectable on physical examination. Although the incidence reaches approximately 20% at 6 weeks when radiological criteria are included, this rate declines markedly to 2.3% by 3 months, supporting the interpretation of seroma as a largely physiological and self-limiting response related to subcutaneous dissection and elevated body mass index rather than a clinically significant complication [11, 25]. Higher body mass index is independently associated with increased radiologically detected seroma, further reinforcing this interpretation. Other complications, such as infection, are mostly managed conservatively, and mesh removal is required in only one case, coinciding with the repair of an incisional hernia, an infrequent complication previously described in other studies [5].

Chronic abdominal wall pain was not systematically assessed beyond 3 months using a validated pain scale. However, routine clinical follow-up included the recording of postoperative events, and no patient required specific treatment or long-term analgesic medication for abdominal wall pain, nor was pain an indication for mesh removal during follow-up.

Although the expected level of radiological follow-up is not achieved, a substantial radiological dataset for assessment of the abdominal wall is obtained, with fewer than 15% of patients lacking imaging at 1 year. Several factors account for this, including the pandemic period, patient refusal to attend follow-up, and the development of additional conditions in this high-risk population. MRI-based analysis indicates no evidence of mesh contraction. More detailed evaluation of the radiological findings in this cohort is currently ongoing [26].

Based on the preventive benefit observed in reducing the incidence of incisional hernia, PMP warrants stronger consideration as part of routine abdominal wall closure in selected high-risk patients, although it is not yet established as the standard of care in international guidelines [6, 13, 27]. Among the available techniques, the onlay position appears particularly appropriate [7, 9, 28], as it combines effectiveness with technical simplicity and broad applicability, making it feasible for surgeons performing abdominal procedures across specialties, including general surgery, vascular surgery, gynecology, and urology, in diverse hospital settings. An additional advantage of this approach is that complications are generally amenable to conservative management, while the retromuscular plane is preserved, thereby maintaining optimal conditions for potential future reconstructive procedures should an incisional hernia eventually develop.

The diagnosis of incisional hernia was based on a standardized assessment combining clinical examination and radiological evaluation, supported by extensive MRI and CT follow-up. Under an intention-to-treat approach, the overall incidence of incisional hernia was 11.3%, a figure that falls within the range reported in previous studies evaluating prophylactic mesh reinforcement [29]. In the per-protocol analysis, excluding patients with early postoperative evisceration, the observed incidence was approximately 9%. Considering that nearly one-quarter of patients underwent contaminated or dirty surgery, one-third underwent emergency procedures, and radiologically detected hernias without clinical relevance were also included, these findings are consistent with those previously reported [5].

Analysis of the data identifies body mass index and stoma formation as factors predisposing to incisional hernia development, findings that are consistent with prior expectations [30–32]. Unexpectedly, longer operative time was associated with a lower incidence of incisional hernia in the multivariable analysis. Given the observational design, the wide range of operative times, and the possibility of residual confounding, this finding should be interpreted with caution and requires confirmation in future studies. Interestingly, three-quarters of the incisional hernias occurred during the first half of the study. Although the study was not designed to evaluate temporal changes in practice, this finding may reflect a more restrictive patient selection during the later stages of recruitment as participating surgeons gained experience with the protocol. Nevertheless, this observation should be interpreted with caution, as other unmeasured factors may also have contributed to the observed difference.

Most patients present with oncologic disease, in addition to other conditions that confer high surgical risk, and are managed in similarly high-risk clinical settings. This profile increases the likelihood of complications and the need for reinterventions, both during the index admission and throughout follow-up. Acceptable long-term outcomes were observed despite the complexity of this patient population.

The debate persists in that, although approximately 85% of surgeons are aware of the benefits of prophylactic mesh, only 3%–15% report routine use [15, 16]. This discrepancy remains difficult to justify, and it underscores the need to critically assess, at the time of abdominal wall closure in high-risk patients, the reasons for not implementing PMP [13, 33], rather than the converse. Hesitation is not limited to surgeons; up to 50% of patients express concerns regarding mesh implantation [14]. When additional factors such as emergency presentation or a cancer diagnosis are present, communication of the potential benefits of PMP becomes even more complex.

In our cohort, patients undergoing emergency surgery or procedures in contaminated fields did not appear to have a higher incidence of mesh-related complications. These findings suggest that prophylactic onlay PVDF mesh reinforcement is feasible in these challenging clinical settings, although confirmation in controlled comparative studies is required. Similarly, the short-term outcomes observed with PVDF mesh in the present study [23], are consistent with those reported in previous studies performed in contaminated surgical fields [34–36].

Among patients undergoing incisional hernia repair, approximately half undergo surgery concurrently for another condition, either for restoration of intestinal continuity or due to oncologic indications, indicating that a second operation would have been required regardless. This argument may be invoked both in favor of and against the use of prophylactic mesh; however, its use is likely to reduce the number of patients requiring subsequent surgery and may also be associated with the development of smaller hernias.

Several limitations should be considered. First, the absence of randomization represents a potential source of bias, including selection bias. Despite clearly defined inclusion criteria, the study period, partly overlapping with the pandemic, and the involvement of multiple centers may have contributed to a relatively limited number of enrolled cases. Furthermore, consecutive inclusion of all eligible patients could not be guaranteed, as patient enrolment also depended on surgeon participation and patient consent. The number and reasons for eligible patients who were not enrolled were not prospectively recorded, limiting the assessment of potential selection bias.

Centre imbalance represents an important limitation. One centre contributed 42.8% of the cohort but accounted for 75% of all incisional hernia events. Because only four events occurred across the remaining centres, centre-adjusted or centre-exclusion sensitivity analyses were considered unreliable. This imbalance may reflect differences in case mix and/or clinical practice and may limit the generalisability of the observed associations. This is particularly relevant to the association between stoma formation and incisional hernia, as stoma formation was also concentrated in the dominant centre.

The multivariable model included four predictors for 16 outcome events (EPV = 4), representing a substantial risk of overfitting and model-selection instability. Although Firth penalised regression and bootstrap internal validation were performed, the resulting associations should be considered exploratory and require confirmation in larger independent cohorts. Ten patients had no available follow-up information and were therefore excluded from the time-to-event analysis, leaving 170 patients for the competing-risks analysis.

In addition, the lack of a standardized fascial closure protocol left an important surgical determinant of incisional hernia uncontrolled, including the choice of suture material, needle type, suture-to-wound length ratio, and the systematic application of the small-bites technique, which may have negatively influenced the study outcomes. MRI compliance was not compared between patients with and without incisional hernia, as a detailed analysis of radiological findings and MRI follow-up is the subject of a separate dedicated manuscript currently in preparation. Moreover, validated patient-reported outcome measures were not collected, precluding a formal assessment of postoperative quality of life and patient-reported symptoms. Furthermore, sex-specific analyses were not performed because the study was not powered for subgroup comparisons according to sex.

The authors’ experience with the mesh and related speaker fees represent a potential source of bias, although these conflicts of interest were fully disclosed. Importantly, most participating surgeons had no conflicts of interest, and all statistical analyses were performed independently by a biostatistician from a public healthcare research institute with no relationship to the supporting company.

Finally, although increasing evidence supports the use of prophylactic mesh in high-risk patients, important questions remain regarding surgical site occurrences, cost–benefit considerations, quality of life, and its role in specific patient populations not represented in the present study, such as those undergoing abdominal aortic aneurysm repair.

Despite these limitations, the study has several important strengths. Its prospective design and the participation of multiple hospitals representing different levels of healthcare, enhancing the generalizability of the findings to a wide range of general surgery departments and clinical scenarios. In addition, the technique evaluated is straightforward and reproducible, even for surgeons not specifically dedicated to abdominal wall surgery. The use of MRI in combination with an iron oxide–loaded PVDF mesh further allows detailed assessment of mesh behavior after implantation. To the authors’ knowledge, no previous prospective multicentre studies have specifically addressed the use of this type of material for onlay prophylactic mesh placement regardless of the level of surgical field contamination.

In conclusion, the present study provides prospective multicentre data on the feasibility of prophylactic onlay PVDF mesh reinforcement in a heterogeneous population of high-risk patients, including emergency and contaminated procedures, with a low observed incidence of incisional hernia and acceptable mesh-related morbidity. These findings should be considered hypothesis-generating and warrant confirmation in future controlled studies.

## Data Availability

The datasets presented in this article are not readily available because they contain patient-level clinical data subject to ethical, institutional, and data protection requirements. Requests to access appropriately de-identified data may be directed to the corresponding author and will be considered subject to approval by the relevant participating institutions and/or ethics committee and compliance with applicable data protection regulations.
